# Influence of polidocanol ultrasound-guided foam sclerotherapy on quality of life in lower extremity chronic venous disease: initial results

**DOI:** 10.1590/1677-5449.190049

**Published:** 2019-10-18

**Authors:** Afonso César Polimanti, Lucas Abdo Pereira, Tainan Montecorado Carmine, Rafael Vilhena de Carvalho Fürst, Alexandre Sacchetti Bezerra, João Antônio Corrêa

**Affiliations:** 1 Faculdade de Medicina do ABC – FMABC, Disciplina de Angiologia e Cirurgia Vascular, Santo André, SP, Brasil.

**Keywords:** prospective studies, quality of life, sclerotherapy/adverse effects/methods, surveys and questionnaires, treatment outcome, venous insufficiency/therapy

## Abstract

Chronic Venous Insufficiency (CVI) is not only detrimental to patients’ Quality of Life (QoL) but also places a considerable burden on public health resources. Ultrasound guided foam sclerotherapy (USFS) is a good treatment option, but its effect on patients’ QOL is still unclear. This article presents the results from the first 27 patients in a prospective, longitudinal, non-controlled study for evaluation of the clinical and QOL impact of USFS treatment for CEAP C4 to C6 grade CVI with contraindications for open surgery. Clinical symptoms were measured with the Venous Clinical Severity Score (VCSS) and QOL by the Assessment of Burden Chronic Disease - Venous questionnaire (ABC-V). We observed 22.2% reductions in VCSS (p<0.001) in the first week after the procedure, and a 37.8% reduction in ABC-V scores (p=0.03) over the first 3 months.

## INTRODUCTION

Chronic venous insufficiency (CVI) is associated with histological and structural changes to the capillary and lymphatic microcirculation, causing multiple physiological disorders, such as capillary extravasation, sequestration of erythrocytes and leukocytes, thrombocytosis, and inflammation of local tissues. The most severe manifestations of this condition and consequent tissue hypoxia include edema of the lower limbs, cutaneous hyperpigmentation, dermatofibrosis, and ulcerations.^1^^-^ 3

Chronic venous ulcers affect millions of people worldwide, constituting a significant cause of reduced quality of life (QoL) and placing a considerable burden on healthcare resources.^4^^-^ 7

According to Brazilian statistical data, CVI is the 14th greatest cause of work absenteeism and the 32nd cause of permanent disability and need for financial support. Surgical treatment is the first choice option for these patients.^5^

Promising results are being reported with alternative methods, including ultrasound guided foam sclerotherapy (USFS), considering the low cost and significant improvements in clinical signs and symptoms of CVI among patients with CEAP C5 and C6, particularly those with contraindications against conventional surgery. Despite the progress with sclerosants for treatment of more advanced grades of CVI, there are still few studies that demonstrate its true impact on patients’ QoL.^4^^-^ 8

The objective of the present study is to present the initial results of an evaluation of the impact of USFS on clinical manifestations and QoL among patients with advanced CVI.

## METHOD

A prospective, longitudinal, uncontrolled study is ongoing to evaluate the impact on clinical manifestations and QoL among patients with advanced CVI.

The study was approved by the Research Ethics Committee at an academic institution, under protocol number CAAE 825697170.0000. The study is partially funded by the Conselho Nacional de Desenvolvimento Científico (CNPq – National Council for Scientific and Technological Development) research funding agency in the form of a research bursary for 2018 and 2019.

The estimated sample size by the end of the study will be 160 patients, but this article was written to present the initial results of the first cases, with 3 months follow-up.

The study recruited patients with a diagnosis of CVI classes CEAP C4 to C6, with visible large-caliber varicose tributaries and/or great saphenous vein insufficiency. Patients were excluded from the study if they had contraindications to USFS, such as a prior thromboembolic event, thrombophlebitis of varicose tributaries, hypersensitivity to polidocanol, or peripheral arterial insufficiency, or if they were pregnant.

Patients who were candidates for the study underwent prospective clinical anamnesis to rule out thrombophilias on the basis of clinical data and to rule out intracardiac shunts using Doppler echocardiography. If there was the slightest suspicion of either of these conditions, the patient in question was excluded from the study.

Patients were given a free and informed consent form, followed by clinical assessment using the Venous Clinical Severity Score (VCSS) and QoL evaluation using the Assessment of Burden Chronic Disease-Venous questionnaire, as validated for Brazilian Portuguese by Almeida et al.^9^ in 2018 (ABC-V).

The foam used during the procedure was produced by mixing polidocanol 3% with air, at a proportion of 1:5, to a total volume of 6 mL. The infusion was administered via a single puncture in a tributary of the leg or directly into the distal great saphenous vein, with the limb elevated, followed by concentric compression with an Unna boot in patients with active ulcers or two 20 to 30 mmHg 7/8 elastic stockings for patients without active ulcers, in addition to lymph myokinetic exercises.

The proportions of the composition and the total volume of foam infused during each session were maintained fixed for all cases in order to avoid confounding bias. All of the vessels treated had a minimum diameter of 5 mm.

Seven days after the procedure, patients underwent clinical reassessment with grading by VCSS and active search for complications using Doppler ultrasonography. Later follow-ups take the form of three-monthly return appointments, with clinical reassessment by VCSS and QoL reassessment with administration of the ABC-V questionnaire after 1 year.

Qualitative variables are expressed as absolute and relative frequencies. Quantitative variables that exhibit normal distribution are expressed as means and standard deviations, while those that do not are expressed as medians and percentiles. Adherence of quantitative variables to the normal distribution is evaluated using the Shapiro Wilk test. The chi-square test is used to analyze associations between qualitative variables. The Mann-Whitney or Student’s *t* tests are used to analyze quantitative variables between groups, depending on the distribution of data. A 95% confidence level is adopted and Stata 11.0 is used for statistical analysis.

## RESULTS

**Figure 1:**
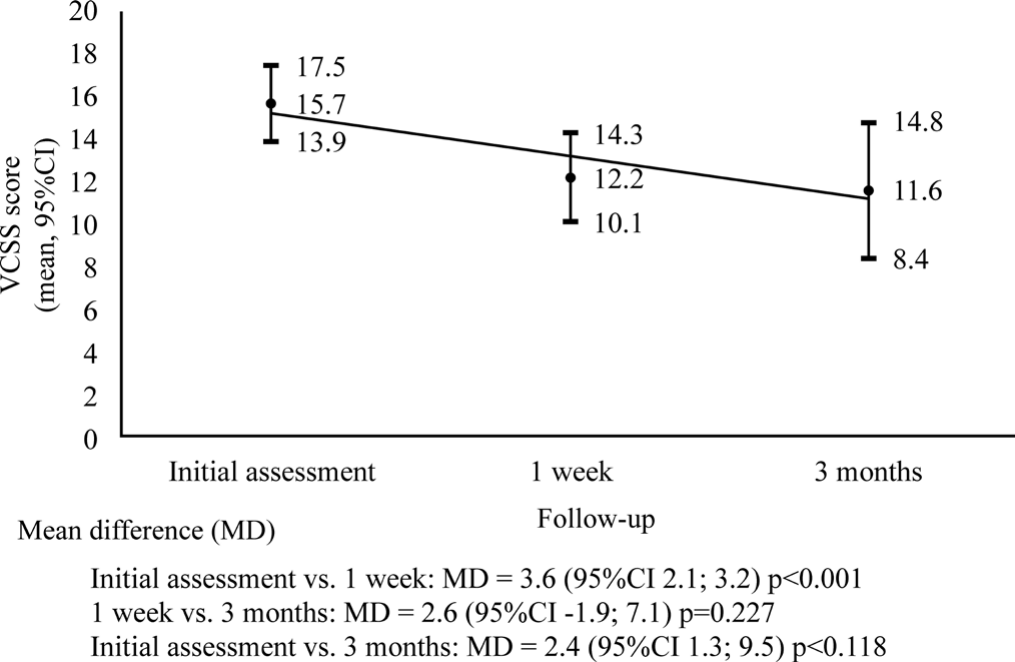
Change in clinical symptomology in the study group over time, according to the VCSS score.

To date, a total of 27 patients have been enrolled on the study, with a total of 36 limbs treated. The VCSS assessments revealed a significant reduction in clinical symptomology (p < 0.001), a mean of 22.2% from the initial assessment to the first week after the procedure and no significant difference (p = 0.227) from the first week to the third month of follow-up, as illustrated by the graph in Figure 1.

**Figure 2:**
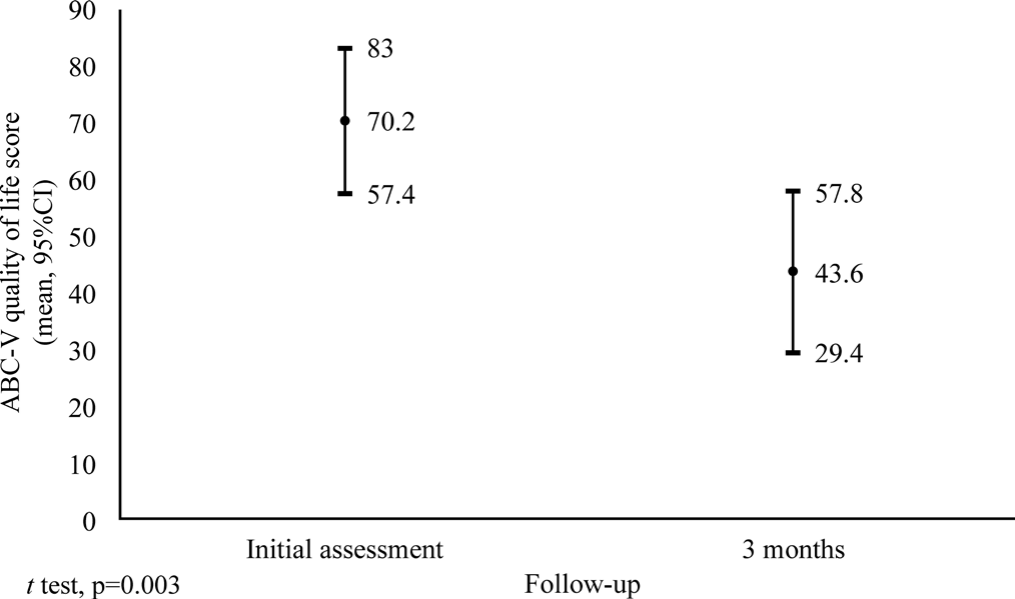
Change in QoL in the study group over time, according to the ABC-V score.

There was also a significant improvement in the quality of life index, demonstrated by a reduction in the ABC-V score from initial assessment to the third month of follow-up. The QoL score at the initial assessment was 70.2, as shown in the graph in Figure 2. There was therefore a significant reduction (p = 0.003) in QoL score, calculated using the ABC-V questionnaire, with a mean reduction of 26.6 points, the equivalent of a 37.8% improvement over the 3-month period.

We have had no adverse events or complications in the 36 limbs treated to date.

## DISCUSSION

Nowadays, CVI is an important cause of work incapacity, demanding heavy public health spending, but despite this it has received little attention in socioeconomic measures.^10^^-^ 15

Over recent decades, hemodynamic studies of people with varicose veins of the lower limbs have demonstrated their relationship with symptoms and trophic lesions over the long term. However, there is a lack of more precise data on the pathogenesis and course of CVI.^10^^-^ 14 ^,^ 16 ^,^ 17

Against this background, many different treatment options have emerged over recent years. One of these, USFS, is an effective option for treatment of CVI with significant reflux, especially in high-risk patients, and offers the advantages of more rapid recovery and the possibility of entirely office-based treatment, significantly reducing the costs involved.^18^

Higher risk patients, who are generally those with contraindications against the conventional surgical procedure, and have more severe cases of CVI, will probably be those who exhibit the greatest impact on QoL, when compared with other groups, and it is exactly this subset that exhibits the greatest benefits when treated with USFS.^19^^,^ 20 In the present study, we have assessed (to date) 36 limbs in 27 patients treated with USFS and our initial results contain data to support this hypothesis.

As illustrated in the graph shown in Figure 1, we observed a reduction in the VCSS score in the first week of follow-up in this sample of patients, with statistical significance, confirming the efficacy of CVI treatment with USFS. These results are similar to those observed in studies by Silva et al.^18^ and Matsuda et al.^21^

In the graph shown in Figure 2, it is possible to observe the impact of treatment on the QoL of the patients involved. The reduction in the ABC-V score shows the efficacy of USFS for significant improvement of patients’ QoL (p = 0.003). These data are compatible with those reported by Campos et al.^5^ and Todd and Wright^22^

The ABC-V questionnaire was described in 2010 by Guex^23^, in France, and later validated in Portuguese by Almeida^9^ in 2018. It was designed to provide a more specific evaluation of the impact of venous symptoms, focusing on six aspects: pain (questions 1 to 4); daily life (questions 5 to 14); interpersonal and family relationships (questions 15 to 18); work (questions 19 to 22); psychological aspects (questions 23 to 32); and perceptions of treatment (questions 33 to 36).^23^ In the initial patients assessed, we did not identify any of these aspects as predominant in terms of impact on QoL.

Although the size of the sample assessed to date is small, it is already possible to identify a significant initial impact of USFS on CVI. We hope that as the study progresses we will be able to collect data to assess duration of remission from symptoms and the rate of maintenance of this increase in patient QoL.

## CONCLUSIONS

The preliminary results show that USFS significantly reduced clinical signs in the first week and was linked with an increase in QoL over the first 3 months in patients with CEAP C4 to C6 grades CVI.
