# Supplementary material for: Influence of polidocanol ultrasound-guided foam sclerotherapy on quality of life in lower extremity chronic venous disease: initial results
Source: J Vasc Bras. 2019 Oct 18;18:e20190049. doi: 10.1590/1677-5449.190049 (PMC6805124; doi:10.1590/1677-5449.190049)
Supplement: Translation

# Translation: Influência da escleroterapia ecoguiada com espuma de polidocanol na qualidade de vida na insuficiência venosa crônica de membros inferiores: resultados iniciais

**Authors:** Afonso César Polimanti, Lucas Abdo Pereira, Tainan Montecorado Carmine, Rafael Vilhena de Carvalho Fürst, Alexandre Sacchetti Bezerra, João Antônio Corrêa

**Affiliations:** 1 Faculdade de Medicina do ABC – FMABC, Disciplina de Angiologia e Cirurgia Vascular, Santo André, SP, Brasil.

**Keywords:** estudos prospectivos, qualidade de vida, escleroterapia/efeitos adversos/métodos, inquéritos e questionários, resultado do tratamento, insuficiência venosa/terapia

## Abstract

A insuficiência venosa crônica (IVC) não só representa um prejuízo na qualidade de vida (QV), como também gera um ônus aos recursos de saúde pública. A escleroterapia ecoguiada com espuma (EEE) vem se mostrando uma boa opção, porém, seu real impacto na QV ainda é incerto. Apresentamos aqui os resultados dos primeiros 27 casos de um estudo prospectivo longitudinal não controlado para avaliação da clínica e QV submetidos a EEE em portadores de IVC CEAP C4 a C6 com contraindicação de cirurgia convencional, com avaliação seriada da clínica por meio do *Venous Clinical Severity Score* (VCSS) e da QV com o questionário *Assessment of Burden Chronic Disease – Venous* (ABC-V). Observamos redução do VCSS de 22,2% (p < 0,001) na primeira semana e do *score* ABC-V de 37,8% (p = 0,003) no primeiro trimestre.

## INTRODUÇÃO

A insuficiência venosa crônica (IVC) está associada a alterações histológicas e estruturais na microcirculação capilar e linfática, produzindo múltiplas alterações fisiológicas, como extravasamento capilar, sequestro de eritrócitos e leucócitos, trombocitose e inflamação dos tecidos locais. As manifestações mais severas dessa condição e da consequente hipóxia tecidual incluem edema de membros inferiores, hiperpigmentação cutânea, dermatofibrose e ulcerações^1^^-^ 3.

A úlcera venosa crônica afeta milhões de indivíduos mundialmente, representando um prejuízo significativo à qualidade de vida (QV), associada também um maior ônus aos recursos de saúde^4^^-^ 7.

Conforme dados estatísticos brasileiros, a IVC representa a 14ª causa de absenteísmo laboral e a 32ª para invalidez permanente e necessidade de assistência financeira. O tratamento cirúrgico nesses pacientes constitui a principal linha terapêutica^5^.

Resultados promissores com métodos alternativos vêm sendo documentados, entre eles a escleroterapia ecoguiada com espuma (EEE), em virtude de seu baixo custo e a significativa melhora dos sinais clínicos e dos sintomas da IVC nos pacientes CEAP C5 e C6, principalmente naqueles com contraindicações à cirurgia convencional. Apesar da evolução dos esclerosantes no tratamento dos graus mais avançados de IVC, ainda há poucos estudos que comprovem o seu real impacto na QV dos pacientes^4^^-^ 8.

O objetivo do presente estudo é apresentar os resultados iniciais da avaliação do impacto da EEE nas manifestações clínicas e na QV de portadores de IVC avançada.

## MÉTODO

Elaborou-se um estudo prospectivo, longitudinal, não controlado, que se encontra em andamento no momento, para a avaliação do impacto nas manifestações clínicas e na QV de pacientes portadores de IVC avançada.

O estudo foi aprovado por Comitê de Ética e Pesquisa de instituição acadêmica, sob o protocolo CAAE: 82569717.0.0000. Houve financiamento parcial do trabalho por parte do Conselho Nacional de Desenvolvimento Científico e Tecnológico (CNPq), sob forma de bolsa de iniciação científica em 2018 e 2019.

A população estimada ao fim do estudo será de 160 pacientes, porém, o presente artigo tem o intuito de apresentar os resultados iniciais, envolvendo os primeiros casos, com seguimento até 3 meses.

Foram incluídos no estudo pacientes com diagnóstico de IVC classes CEAP C4 à C6, com aparentes tributárias varicosas de grande calibre e/ou insuficiência de veia safena magna. Excluíram-se do estudo todos aqueles pacientes apresentando contraindicações à EEE, como evento tromboembólico prévio, tromboflebite de tributárias varicosas, hipersensibilidade ao polidocanol, gestação em curso ou insuficiência arterial periférica.

Os pacientes candidatos ao estudo foram submetidos à pesquisa ativa diagnóstica de trombofilias através de dados de anamnese clínica, e de *shunts* intracardíacos por meio de estudo ecocardiográfico com Doppler. Na mínima suspeita de alguma dessas afecções, o paciente em questão era excluído do estudo.

Aplicou-se um termo de consentimento livre esclarecido, seguido de avaliação clínica por meio do *Venous Clinical Severity Score* (VCSS) e de QV com o questionário *Assessment of Burden Chronic Disease-Venous* validado para o português brasileiro por Almeida et al.^9^ em 2018 (ABC-V).

No procedimento, a espuma foi obtida por meio da mistura de polidocanol 3% com ar, na proporção de 1:5, totalizando volume de 6 mL. Realizou-se a infusão em punção única em tributária de perna ou diretamente em veia safena magna distal, com membro elevado, seguida de compressão concêntrica com bota de Unna nos pacientes com úlcera ativa ou associação de duas meias elásticas 7/8 de 20 a 30 mmHg nos pacientes sem lesão ativa, com realização de exercícios linfomiocinéticos.

A proporção da composição da espuma e volume total infundido na sessão foram mantidos fixos em todos os casos no intuito de evitar viés de confusão nos dados obtidos. Todos os vasos abordados tinham diâmetro mínimo de 5 mm.

Sete dias após o procedimento, os pacientes foram submetidos a reavaliação clínica com novo estadiamento com VCSS e pesquisa ativa de complicações com ultrassonografia com Doppler. O seguimento mais tardio é realizado por meio de retornos trimestrais, com reavaliação clínica com VCSS e de QV com reaplicação do questionário ABC-V pelo período de 1 ano.

As variáveis qualitativas estão sendo descritas por frequência absoluta e relativa, respectivamente. As variáveis quantitativas que apresentam aderência à distribuição normal estão sendo descritas por médias e desvios padrão, enquanto aquelas que não apresentam essas características estão sendo descritas por medianas e percentis. A aderência das variáveis quantitativas à distribuição normal está sendo avaliada pelo teste de Shapiro Wilk. O qui-quadrado está sendo utilizado para analisar a associação entre variáveis qualitativas. O Mann-Whitney ou teste t de student estão sendo utilizados para analisar as variáveis quantitativas entre os grupos, de acordo com a distribuição dos dados. O nível de confiança adotado é de 95%. O programa utilizado para análise estatística é o Stata 11.0.

## RESULTADOS

Até o momento, foram incluídos no estudo 27 pacientes, totalizando 36 membros abordados. Observou-se, na avaliação do VCSS, uma redução significante da sintomatologia clínica (p < 0,001) de em média 22,2% da avaliação inicial para a primeira semana pós-procedimento, sem diferença significativa (p = 0,227) da primeira semana para o terceiro mês de seguimento, como mostra o gráfico da Figura 1.

**Figura 1:** Variação da sintomatologia clínica por meio do *score* VCSS no grupo de estudo ao longo do tempo.

Houve também melhora significativa nos índices de qualidade vida, demonstrado pela redução do *score* ABC-V entre a avaliação inicial e o terceiro mês de seguimento. O *score* de QV da avaliação inicial foi de 70,2, como mostra o gráfico da Figura 2. Dessa forma, houve uma redução significativa (p = 0,003) do *score* de QV, de acordo com o questionário ABC-V, com decréscimo em média de 26,6 pontos no *score*, representando uma melhora de 37,8% no período de 3 meses.

**Figura 2:** Variação da QV por meio do *score* ABC-V no grupo de estudo ao longo do tempo.

Até o presente momento, não tivemos nenhum evento adverso ou complicação nos 36 membros abordados.

## DISCUSSÃO

A IVC é hoje uma importante causa de incapacidade laboral, promovendo um vultoso gasto na saúde pública; apesar disso, ela tem sido pouco abordada em medidas socioeconômicas^10^^-^ 15.

Ao longo das últimas décadas, estudos hemodinâmicos de portadores de varizes de membros inferiores mostram sua relação com os sintomas e alterações tróficas a longo prazo. Porém, dados mais concisos sobre patogenia quanto à progressão precisa da IVC permanecem incertos^10^^-^ 14 ^,^ 16 ^,^ 17.

Nesse contexto, diversas opções terapêuticas têm-se mostrado nos últimos anos. Entre elas, a EEE constitui uma alternativa eficaz para o tratamento da IVC com refluxo significativo, em especial nos pacientes de alto risco, apresentando ainda vantagens de uma recuperação mais rápida e a possibilidade de tratamento completamente ambulatorial, reduzindo significativamente o custo^18^.

Os pacientes de maior risco, que geralmente apresentam contraindicações ao procedimento cirúrgico convencional, com quadros mais graves de IVC, muito provavelmente serão os que apresentam maior impacto na QV quando comparados a outros grupos, e são exatamente esses os que apresentam maior benefício quando submetidos à EEE^19^^,^ 20. No presente estudo, avaliamos, até o momento, 36 membros em 27 pacientes submetidos à EEE e encontramos, em nossos resultados iniciais, dados a favor dessa hipótese.

Conforme apresentado no gráfico da Figura 1, observamos uma redução do *score* VCSS na primeira semana de seguimento desses pacientes, com relevância estatística, comprovando a eficácia no tratamento da IVC por meio da EEE. Esses resultados são semelhantes aos estudos de Silva et al.^18^ e Matsuda et al.^21^

No gráfico da Figura 2, observamos a o impacto do tratamento na QV dos pacientes envolvidos. A redução do score ABC-V mostra que a EEE é eficaz na melhora a QV do paciente de forma significativa (p = 0,003), dados compatíveis aos apresentados por Campos et al.^5^ e Todd e Wright.^22^

O questionário ABC-V, descrito em 2010 por Guex^23^, na França, e posteriormente validado ao português por Almeida^9^, em 2018, foi concebido com o intuito de avaliar de forma mais específica o impacto dos sintomas venosos, com enfoque em seis aspectos: dor (questões 1 a 4); vida diária (questões 5 a 14); relacionamento interpessoal e familiar (questões 15 a 18); laboral (questões 19 a 22); psicológico (questões 23 a 32); percepção do tratamento instituído (questões 33 a 36).^23^ Não identificamos, na casuística inicial, uma preponderância de nenhum desses aspectos no impacto da QV em nosso estudo.

Apesar de, no momento, o tamanho da amostra ainda ser reduzido, já é possível identificar o impacto inicial significativo da EEE na IVC. Esperamos, com a evolução do estudo, obter dados para avaliar o tempo de remissão dos sintomas e a taxa de manutenção deste incremento na QV dos pacientes.

## CONCLUSÃO

Os resultados preliminares mostram que a EEE reduz significativamente a clínica na primeira semana, bem como traz um incremento à QV nos primeiros 3 meses em portadores de IVC CEAP C4 a C6.
